# Public policies, State and prevention of skin cancer related to occupational exposure: an integrative review

**DOI:** 10.47626/1679-4435-2025-1477

**Published:** 2025-09-14

**Authors:** Kaia Aparecida Nunes Faria Gomes, Nicolly Teixeira de Oliveira, Ana Carolina Hartwig Pereira, Italla Maria Pinheiro Bezerra

**Affiliations:** 1 Master’s in Public Policy and Local Development, Graduate Department, Escola Superior de Ciências da Santa Casa de Misericórdia (Emescam), Vitória, ES, Brazil.; 2 Bachelor’s in Nursing, Emescam, Vitória, ES, Brazil

**Keywords:** skin neoplasms, health policy, ultraviolet rays, occupational exposure, neoplasias cutâneas, política de saúde, raios ultravioleta, exposição ocupacional

## Abstract

Currently, skin cancer is the most common among all cancer types. Despite its high incidence, the topic is rarely discussed, as few cases result in death. The aim of the current study is to describe public policies and the role of the State in ensuring care and preventing skin cancer related to occupational exposure. This is an integrative review conducted between May and July 2024 in the Latin American and Caribbean Literature on Health Sciences (LILACS), MEDLINE/PubMed, and Cochrane electronic databases. The search strategy employed in the databases used the following English-language descriptors, recognized by the Health Sciences Descriptors system: (“skin neoplasms” OR “melanoma” OR “basal cell carcinoma” OR “squamous cell carcinoma”) AND (“health policy” OR “labor legislation” OR “human rights”) AND (“ultraviolet rays” OR “occupational exposure”). Twenty articles were selected, with most studies (n = 12) discussing the role of public policies in the context of skin cancer in workers exposed to radiation. Although there have been advances in workplace sun safety policies, gaps remain in their effectiveness, and it is necessary to invest in training, communication, and international references to improve them.

## Abstract (pt)

Atualmente, o câncer de pele é o mais frequente entre todos os tipos de câncer. Apesar de sua alta incidência, discute-se pouco sobre o tema, pois poucos casos levam a óbito. O objetivo deste estudo é descrever sobre as políticas públicas e o papel do Estado na garantia da assistência e na prevenção do câncer de pele relacionado à exposição ocupacional. Trata-se de uma revisão integrativa, realizada entre os meses de maio e julho de 2024, nas bases eletrônicas da Literatura Latino-Americano e do Caribe em Ciências da Saúde (LILACS), MEDLINE/PubMed. A estratégia de busca empregada nas bases de dados utilizou os seguintes descritores na língua inglesa, reconhecidos pelo sistema de Descritores em Ciências da Saúde: (“neoplasias cutâneas” OR “melanoma” OR “carcinoma basocelular” OR “carcinoma espinocelular”) AND (“política de saúde” OR “legislação trabalhista” OR “direitos humanos”) AND (“raios ultravioleta” OR “exposição ocupacional”). Foram selecionados 20 artigos, sendo que a maioria das pesquisas (n = 12) apresentou discussões sobre o papel das políticas públicas no contexto do câncer de pele em trabalhadores expostos à radiação. Embora haja avanços nas políticas de segurança solar no trabalho, ainda existem falhas em sua eficácia, sendo necessário investir em formação, comunicação e referências internacionais para aprimorá-las.

## Introduction

Cancer is a disease characterized by disorganized cell growth, with the ability to invade other tissues, cause metastasis, and ultimately lead to death. Among the different types, skin cancer is the most common worldwide. Of the various forms of skin cancer, basal cell carcinoma and squamous cell carcinoma are the most frequent, whereas melanoma, although less common, is the most aggressive and lethal. The primary risk factor for the development of skin cancer is exposure to ultraviolet radiation (UVR).^1^ Therefore, individuals most exposed to UVR — whether for leisure or work-related reasons — are more vulnerable to developing skin cancer.

Global data on occupational UVR exposure indicate that 28% of the economically active population is exposed. Among these individuals, approximately 33% of deaths attributable to skin cancer are associated with occupational sun exposure, most occurring among men and older adults. Therefore, occupational exposure to solar UVR is one of the main work-related risk factors for cancer worldwide.^2^

The preventive measures recommended by the World Health Organization (WHO) and the International Labour Organization (ILO) include joint actions among governments, employers, and workers, such as providing personal protective equipment (PPE), adjusting working hours to avoid exposure during peak UVR periods, ensuring shaded areas in outdoor workplaces, and offering education and training on the risks of excessive sun exposure and the importance of photoprotection. As a supporting tool, the SunSmart Global UV App was launched by the WHO, ILO, World Meteorological Organization, and United Nations Environment Programme, providing UVR index forecasts and personalized guidance for the public.^3^

In Brazil, since 2014, the Brazilian Society of Dermatology (Sociedade Brasileira de Dermatologia, SBD), in partnership with the government, has developed the annual “Dezembro Laranja” (Orange December) campaign, aimed at raising awareness about skin cancer prevention. Specifically for outdoor workers, in 2024, the SBD supported the inclusion of sunscreen in the list of essential items in Brazil’s Tax Reform, seeking to reduce taxes on these products and improve access to sun protection. The provision of PPE, such as hats and sunscreen, for outdoor workers by companies is part of the labor legislation established in Law No. 8,231/91.^4^

In 2017, Moura et al.^5^ conducted a study addressing skin cancer as a public health issue. The study showed that Brazil has various laws guaranteed by the Federal Constitution, including the population’s right to health care at all levels of complexity. One of the greatest achievements of the Brazilian health care system is Law No. 8,080, which regulates the organization of health services and the promotion, protection, and recovery of health. Most health problems and the prevention of many diseases, including skin cancer, could be addressed with a well-structured primary health care system.

Because of the importance of prevention and early diagnosis of occupational skin cancer, a literature review was conducted focusing on the role of occupational physicians. The aim of that work was to guide occupational physicians on the subject and improve disease prevention strategies. The study highlighted that the role of the occupational physician is essential for raising awareness and disseminating information about skin cancer and its different forms of prevention, as well as for early diagnosis and reporting of work-related cases.^6^

Thus, although the prevalence of UVR exposure among Brazilian workers is high, there is inequality between public and private health care. The State has the obligation to provide all individuals vulnerable to developing skin cancer for work-related reasons with adequate care — from primary to tertiary levels — covering prevention, health promotion, control, treatment, and rehabilitation. Therefore, this study aimed to describe public policies and the role of the State in ensuring care for preventing occupational exposure-related skin cancer.

## Methods

### Type of study

This is an integrative review aimed at compiling and synthesizing the results of scientific publications related to human rights and public policies in the prevention and treatment of skin cancer in occupational exposure contexts.

The integrative review is the broadest methodological approach among review types, constituting a study that combines the analysis of multiple published studies with different methodologies, with the purpose of gaining a deep understanding of a given phenomenon based on previous research.

### Stages of the integrative review

This review was conducted in stages, adapted from the Preferred Reporting Items for Systematic Reviews and Meta-Analyses (PRISMA) recommendations. The process included: identification of subject and selection of the review question for integrative review; establishment of inclusion and exclusion criteria; definition of the information to be extracted from selected studies to enable categorization of findings; evaluation of included studies; interpretation of results; and preparation of review/knowledge synthesis presentation.^7^

### Review question

What is the role of State and public policies in the prevention of skin cancer related to occupational exposure?

### Eligibility criteria

All scientific publications addressing public health, skin cancer, and occupational exposure were considered. No restriction was imposed to year of publication due to the scarcity of recent articles on the subject. Articles in English and Portuguese were included regardless of study design and methodological quality. Exclusion criteria were abstracts published in journals or conference proceedings, duplicate publications across the databases explored, theses, and dissertations.

### Information sources and search strategy

The literature search was conducted between May and July 2024 in the Latin American and Caribbean Literature on Health Sciences (LILACS), MEDLINE/PubMed, and Cochrane electronic databases.

The search strategy used in these databases included the following English-language descriptors, recognized by the Health Sciences Descriptors system: (“Skin Neoplasms” OR “Melanoma” OR “Carcinoma basal cell” OR “Carcinoma squamous cell”) AND (“Health policy” OR “Legislation labor” OR “Human right”) AND (“Ultraviolet rays” OR “Occupational exposure”).

The search strategies used are presented in Table 1.

**Table 1:** Study objective and data extraction

Objective/problem	What is the role of State and public policies in the prevention of skin cancer related to occupational exposure?		
	P	I	Co
Extraction	Neoplasias cutâneas Melanoma Carcinoma basocelular Carcinoma espinocelular	Política de saúde Legislação trabalhista Direitos humanos	Radiação ultravioleta Exposição ocupacional
Conversion	Skin Neoplasms Melanoma Carcinoma, basal cell Carcinoma, squamous cell	Health policy Legislation, labor Human rights	Ultraviolet rays Occupational exposure
Construction	“Skin Neoplasms” OR “Melanoma” OR “Carcinoma, basal cell” OR “Carcinoma, squamous cell”	“Health policy” OR “Legislation, labor” OR “Human right”	“Ultraviolet rays” OR “Occupational exposure”
Application	(“Skin Neoplasms” OR “Melanoma” OR “Carcinoma basal cell” OR “Carcinoma squamous cell”) AND (“Health policy” OR “Legislation labor” OR “Human right”) AND (“Ultraviolet rays” OR “Occupational exposure”)		

### Study selection

Initially, the identified studies were evaluated by analyzing their titles, excluding duplicates across the databases as well as those unrelated to the aims of the current study. Next, abstracts were read, and those that did not meet the predefined inclusion criteria were excluded. Finally, full-text reading was performed to select studies aligned with the aims of this review, resulting in the final sample included in this integrative review.

All studies identified through the search strategy were first screened by title and abstract, according to the eligibility criteria. Those meeting the requirements to answer this review question were selected.

**Figure 1.**
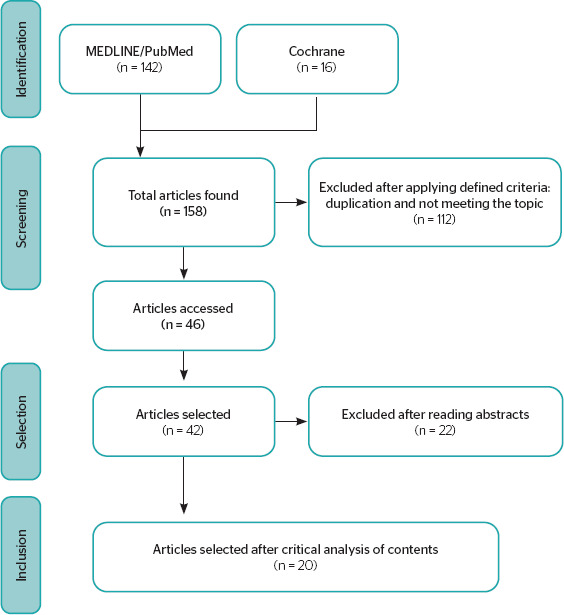
Preferred Reporting Items for Systematic Reviews and Meta-Analyses (PRISMA) flowchart.

Study selection was performed initially by a single review author, with any uncertainties resolved in consultation with a second one. The whole study selection process, including the reasons for and number of exclusions at each stage, is summarized in the flowchart shown in Figure 1, adapted from PRISMA recommendations.^7^

LILACS = Latin American and Caribbean Literature on Health Sciences.

### Data/content extraction and analysis

In order to characterize the articles included in the review, the following information was extracted: title, author/year, type of study, and objectives. To further explore the focus of this review, additional information was collected on the study objective, method, conclusion, and novelty.

To compile and present the results regarding challenges, strategies, and proposals, the discursive textual analysis technique proposed by Moraes & Galiazzi^8^ was applied. Based on the aims of this review, collected data were initially analyzed and divided into units of meaning, and subsequently grouped by similarity, allowing for the establishment of the analysis categories that will be discussed in the third section of this study.

## Results

A total of 158 publications were identified in the databases, 16 from Cochrane and 142 from MEDLINE/PubMed. After removing duplicate studies, 46 publications remained for evaluation according to the defined parameters. Of these, 20 met the inclusion criteria and contained the necessary elements to answer the proposed guiding question. The whole selection process that led to the inclusion of these studies is presented in the flowchart in Figure 1.

Analysis of the characteristics of the studies selected for this review revealed considerable variation in year of publication, with the oldest dating from 2004 and the most recent from 2024. Most studies (n = 12) discussed the role of public policies in the context of skin cancer among workers exposed to UVR (Table 2).

**Table 2:** Characteristics of selected studies

Author/year	Title	Novelty
Conte et al.,^12^ 2023	Skin Cancer Prevention across the G7, Australia and New Zealand: A Review of Legislation and Guidelines	Based on the information gathered, recommendations were developed to help reduce skin cancer rates in the coming years, following the successful models of countries such as Australia and New Zealand.
Mirza et al.,^11^ 2023	Considering Sun Safety Policies in the United States	These comparative examples can assist in interventions in the USA and have the potential to modify exposure to skin cancer-related risk factors.
Peters et al.,^15^ 2019	Burden of non-melanoma skin cancer attributable to occupational sun exposure in Canada	In 2011, 6.31% (4,556 cases) of non-melanoma skin cancer cases were estimated to be attributable to occupational UVR exposure. Most of these cases occurred in men working in agriculture or construction.
Yu et al.,^25^ 2023	Risk factors and early prevention of skin cancer in rural older outdoor workers: A scoping review	Highlights the importance of developing feasible preventive strategies to reduce disparities in skin cancer care and access to preventive services among rural populations at high risk for developing this type of cancer. Further investigations and policy actions are needed.
Edlich et al.,^10^ 2004	National health strategies to reduce sun exposure in Australia and the United States	Australia has adopted a more effective approach to skin cancer prevention, making UVR protection a social responsibility through massive educational campaigns, strict regulation of sun protection products, and legislative initiatives.
Elsner et al.,^17^ 2013	UV-induced occupational skin cancer: possibilities of secondary individual prevention in the “Dermatologist’s Procedure”	Based on this agreement, the Social Accident Insurance has the tools to independently provide preventive measures for the new occupational skin disease, SCC caused by natural UVR, in accordance with §9 Section 2 of the German Social Code (Sozialgesetzbuch, SGB) VII.
Taber et al.,^14^ 2018	Skin cancer interventions across the cancer control continuum: Review of technology, environment, and theory	The results suggest that skin cancer-specific technology and environmental modifications are underused in skin cancer behavioral interventions.
Tran et al.,^28^ 2022	The Value of Partnerships in Multi-Component Skin Cancer Prevention Interventions	Rhode Island developed a coordinated statewide partnership among stakeholders, providing valuable resources and expertise, maximizing the reach and effectiveness of targeted skin cancer prevention and screening programs.
Wittlich,^16^ 2022	Criteria for Occupational Health Prevention for Solar UVR Exposed Outdoor Workers-Prevalence, Affected Parties, and Occupational Disease	Most occupations and sub-occupations investigated so far exceed the threshold for providing occupational health care.
Glanz et al.,^18^ 2007	Reducing ultraviolet radiation exposure among outdoor workers: state of the evidence and recommendations	Few pieces of evidence were found in the reports to recommend current strategies as effective.
Eide & Weinstock,^27^ 2006	Public health challenges in sun protection	Public health efforts have established a solid foundation for promoting and developing effective health campaigns and policies that encourage sunscreen use and primary skin cancer prevention.
Pil et al.,^29^ 2016	Burden of skin cancer in Belgium and cost-effectiveness of primary prevention by reducing ultraviolet exposure	Provided an accurate estimate of the current and future impact of skin cancer in Belgium. Demonstrated that a population-based national strategy promoting UVR protection behavior can bring health and economic benefits for health care payers as well as from a societal perspective.
Lazovich et al.,^24^ 2012	Time to get serious about skin cancer prevention	Despite progress made in reducing the burden of common cancers in the USA, it is now time to take seriously the cancer that affects more Americans than all other cancers combined.
Walkosz et al.,^20^ 2019	Senior managers’ awareness of sun protection policy predicts implementation of worksite sun safety in a randomized trial	There was an increase in communication about sun safety among employees, in the availability of sun protection items at the workplace, and in senior managers’ awareness of existing sun safety policies.
Glanz et al.,^23^ 2022	Development of a Survey of Sunscreen Use and Attitudes among Adults in Two Coastal States	The survey showed good test-retest reliability, reasonable knowledge and attitudes about sunscreen use, and awareness of the law, although without familiarity with its main features.
Walkosz et al.,^19^ 2018	Sun Safe Workplaces: Effect of an Occupational Skin Cancer Prevention Program on Employee Sun Safety Practices	Occupational skin cancer prevention should be addressed through the promotion of targeted policies.
Nahar et al.,^13^ 2019	Sun protection behaviors of state park workers in the Southeastern USA	Most state park workers did not display adequate sun protection behaviors, with sunglasses being the most frequently used protective equipment.
Woolley et al.,^22^ 2008	Workplace sun protection policies and employees’ sun-related skin damage	Employees working under mandatory sun protection policies had less sun-related skin damage, likely due to reduced sun exposure.
Hall et al.,^21^ 2009	Lifeguards’ sun protection habits and sunburns: association with sun-safe environments and skin cancer prevention program participation	There was a trend toward fewer sunburns as social norms, pool policies, and participation in the Pool Cool program increased, although results varied over the 2 years.
Symanzik & John,^26^ 2024	Prevention of occupational skin cancer caused by Solar UVR exposure: recent achievements and perspectives	Existing strategies for the prevention of occupational skin cancer can be improved not only in the general and individual prevention components but also in environment- and behavior-based prevention.

## Discussion

Health imbalance can lead to the development of numerous diseases. However, health, in its broadest sense, goes beyond the concept adopted at the 8th National Health Conference in 1986, which defines it as physical, emotional, and social well-being.^9^ The purpose of this study was to examine the literature on the relationship between human rights and public policies related to skin cancer caused by occupational exposure.

The selected studies address sun protection interventions and policies aimed at reducing the risk of developing skin cancer among outdoor workers. They also highlight the importance of structured educational programs, community involvement, and workplace policies to promote photoprotective behaviors. Despite some positive trends, global evidence remains inconsistent, with many studies presenting limitations such as reliance on self-reported measures and insufficient demographic data. This reinforces the need for more effective regulations and comprehensive public health strategies to improve sun safety practices and reduce the incidence of skin cancer.

A total of 3 articles were identified that compared different countries. The oldest showed that Australia has broader preventive measures for skin cancer — including specific sunscreen legislation and educational campaigns — whereas in the USA, responsibility tends to be placed on the individual.^10^ Another comparative study involving Australia, the USA, and the UK highlighted shortcomings in USA sun protection policies, including the lack of adequate sunscreen regulation and the absence of a ban on tanning bed use.^11^ A broader review included G7 countries, Australia, and New Zealand, showing that, in the workplace, with the exception of Australia and some jurisdictions in the USA, protection against UVR is poor and lacks comprehensive regulation.

In France, Italy, Germany, the UK, and Japan, only ionizing or artificial radiation is recognized as a physical hazard and therefore addressed within Occupational Health and Safety.^12^ The predominance of Australian studies reflects that country’s early interest in skin cancer prevention since its high incidence and mortality rates.

Most of the articles reviewed originated from the USA and, regardless of the methodology used, reported similar findings, revealing shortcomings in policies aimed at preventing skin cancer. Among these, 2 studies stand out for assessing behavioral sun protection measures among outdoor workers.

A study of state park workers in the southwestern USA, based on the Health Belief Model (HBM), found that approximately 52.3% never or rarely used sunscreen.^13^ Another study, which reviewed 86 articles published in 2000-2015 on the use of technology, environmental modifications, and theoretical frameworks in skin cancer prevention and control, found that interventions via mobile devices can lead to self-reported behavior changes. Enhancing the personalization and targeting of messages was recently identified by an expert panel as a relevant topic for sun safety, with technology offering the potential to deliver these tailored messages to large population groups.^14^

In a study conducted in Canada, the authors concluded that 6.31% of nonmelanoma skin cancer cases may be related to occupational sun exposure, particularly in the agricultural and construction sectors. To mitigate these risks, the authors recommend implementing workplace safety policies.^15^ In Germany, another study showed that approximately 87% of outdoor worker occupations analyzed exceeded adequate exposure levels.^16^ Focusing on secondary prevention for sun-exposed workers, this study recognizes squamous cell carcinoma as an occupational disease and reinforces the need for wearing photoprotective clothing and adopting shaded breaks during peak UVR hours.^17^

A study conducted in the USA evaluated knowledge, attitudes, and public policies related to sun exposure and protection among outdoor workers. Results indicated that women tend to use sunscreen more frequently, while the use of hats and protective clothing is more common among men.^18^

Other USA-based studies using targeted programs for outdoor workers in their workplaces reported varied results. In 2018, Walkosz et al.^19^ found that in workplaces where formal policies were adopted, there was a relevant increase in the use of sunscreen and the wearing of PPE. In 2019, in a randomized controlled trial, the same author concluded that manager awareness positively influences the implementation of sun protection measures, such as providing PPE and actively communicating the issue to workers.^20^

In another cross-sectional study, researchers assessed social norms, safety policies, and participation in the Pool Cool skin cancer prevention program. Results indicated that more frequent sunscreen use and fewer sunburns among participants were associated with the presence of adequate institutional policies and social norms favorable to photoprotection.^21^

Similarly, another study showed that when a sun protection policy exists — including providing sunscreen and mandating PPE use — there is a significant reduction in skin damage among outdoor workers.^22^ Studies evaluating adults’ sunscreen use and attitudes revealed that sunscreen use was higher mainly among older adults and women; however, knowledge about ingredients and legislation was low.^23^

Several articles highlight the ineffectiveness of current sun protection strategies, the inconsistency of institutional recommendations, and the lack of investment in ongoing educational programs, even amid rising skin cancer incidence rates.^24^ From the perspective of skin cancer prevention strategies, a scoping review involving older rural outdoor workers identified prolonged exposure to UVR as the main risk factor. It also pointed out that limited access to preventive measures and early diagnosis exacerbates the vulnerability of this group.^25^

A recent study assessed preventive strategies classified into technical, organizational, and individual measures. Outdoor workers, such as farmers and construction workers, were identified as the most affected by exposure to UVR. The study emphasizes the importance of stricter regulations for these groups, which highlights the need for educational actions and the implementation of effective public policies.^26^

Another article identifies UVR as the main modifiable factor in the development of skin cancer — especially melanoma, whose incidence has been rising in recent decades. It also notes that irregular sunscreen use significantly reduces product effectiveness and addresses the “compensation hypothesis,” which links sunscreen use to longer sun exposure. The authors recommend promoting educational campaigns on safe sun behaviors and encouraging the combined use of protective clothing, hats, and shade.^27^

A study conducted in Rhode Island, USA, highlights the importance of public-private partnerships in community-based actions for skin cancer prevention. It also emphasizes the relevance of educational campaigns, especially those that combine free screenings at beaches and parks with sunscreen distribution. This integration between health agencies, academic institutions, and media companies promotes greater adherence to sun protection and, consequently, contributes to reducing the incidence of skin cancer.^28^

In Belgium, one of the few studies evaluating the economic impact of skin cancer was carried out. Projections indicate that the disease’s prevalence will triple in the next 20 years, resulting in an accumulated cost of €3 billion, with melanoma accounting for most of these expenses. To reduce incidence and generate savings for the health system, the authors recommend intensifying awareness campaigns. They stress that, to achieve this goal, public policies restricting exposure to UVR play a fundamental role.^29^

Despite methodological limitations, such as selection bias and database restrictions, there is a clear need to invest in education and public policies aimed at expanding knowledge about skin cancer, thereby raising awareness among sun-exposed workers on the importance of behavioral change regarding photoprotection.

Within this context, it is important to recall the role of the State in ensuring the health of every citizen. According to Article 3 of Topic 1 of the final report from the 1986 National Health Conference^9^:

The right to health means the guarantee, by the State, of dignified living conditions and universal and equal access to actions and services for health promotion, protection, and recovery, at all levels, for all inhabitants of the national territory, leading to the full development of the human being as an individual [free translation].

Specifically regarding occupational skin cancer, the guidelines for surveillance of work-related cancers have expanded visibility on the subject. The lack of information and scarcity of national research on work-related cancers are evident when considering that, among work-related sick-leave benefits granted by Social Security, only 749 cases (0.23%) were attributed to neoplasms related to work.^30^ Nonetheless, the State plays a fundamental role in protecting workers, ensuring safe working conditions, promoting awareness campaigns, and monitoring compliance with occupational health regulations. Preventing exposure, ensuring early diagnosis, and providing appropriate treatment for identified cases are part of labor legislation, helping to reduce the disease’s impact on the economically active population.^31^

Implementing specific public policies for occupational skin cancer is essential. Worker health programs should include educational actions on photoprotection, the provision of adequate PPE, and periodic examinations for the early detection of suspicious lesions.^32^ To ensure dignity and the right to health in accordance with the Brazilian Federal Constitution, compensation and social support policies for workers affected by occupational cancer should be adjusted, for example, through investments in epidemiological surveillance and research.^33^

Therefore, State action in the prevention and management of occupational skin cancer must be integrated and continuous, encompassing everything from workplace regulation to health promotion and social assistance. Without a strategic and intersectoral approach, vulnerable workers will remain exposed to avoidable risks, perpetuating inequalities and significant economic and social impacts for the country.

## Conclusions

Although workplace sun safety policies show positive trends in terms of sunscreen use and the creation of an environment conducive to sun protection, significant gaps remain in their effectiveness and implementation. In order to increase the impact of these policies, it is recommended that governmental organizations adopt more comprehensive sun safety training, improve communication on the subject, and draw lessons from successful international models.
